# Synthetic biology meets tissue engineering

**DOI:** 10.1042/BST20150289

**Published:** 2016-06-09

**Authors:** Jamie A. Davies, Elise Cachat

**Affiliations:** * Centre for Integrative Physiology and Synthsys Mammalian, University of Edinburgh, Edinburgh EH8 9XB, U.K.

**Keywords:** development, morphogenesis, self-organization, synthetic biology, synthetic morphology, tissue engineering

## Abstract

Classical tissue engineering is aimed mainly at producing anatomically and physiologically realistic replacements for normal human tissues. It is done either by encouraging cellular colonization of manufactured matrices or cellular recolonization of decellularized natural extracellular matrices from donor organs, or by allowing cells to self-organize into organs as they do during fetal life. For repair of normal bodies, this will be adequate but there are reasons for making unusual, non-evolved tissues (repair of unusual bodies, interface to electromechanical prostheses, incorporating living cells into life-support machines). Synthetic biology is aimed mainly at engineering cells so that they can perform custom functions: applying synthetic biological approaches to tissue engineering may be one way of engineering custom structures. In this article, we outline the ‘embryological cycle’ of patterning, differentiation and morphogenesis and review progress that has been made in constructing synthetic biological systems to reproduce these processes in new ways. The state-of-the-art remains a long way from making truly synthetic tissues, but there are now at least foundations for future work.

## Introduction

Synthetic biology has so far been dominated by systems that operate at the single-cell level. Much engineering work, for example constructing modules for new metabolism or biosensing, has been done in single-celled microorganisms with little or no possibility for multicellular organization (examples may be found in [1–3]) and, even where host cells from multicellular organisms such as animals and plants have been used, multicellularity has usually been irrelevant to the aims of the project. A few projects have, however, focused on the behaviour of cell collectives, for example to synchronize synthetic oscillators [4] or to orchestrate simple multicellular morphogenetic events [5]. These projects have demonstrated the feasibility of constructing mechanisms that use cooperative actions of engineered cells and, we argue in this article, open the door to the application of synthetic biological techniques to the challenges of advanced tissue engineering.

## Conventional tissue engineering

Tissue engineering is a multidisciplinary endeavour and its aim is to construct or reconstruct tissues for the repair of bodies that are damaged or in some way unusual [6]. Its challenges vary in scale from construction of relatively simple tissues to complex organs. An example of the relatively simple is cartilage, an avascular tissue that contains few cell types and a relatively homogeneous matrix, and which can be valuable to surgeons for engraftment into damaged joints [7]. An example of the very complex is the kidney, a highly complex system of fine tubes and filters arranged with great precision with respect to one another and with respect to vasculature, and that contains at least 60 distinct tissue types [8]. Cartilage engineering has reached a stage of maturity that has seen direct clinical application [7]. Kidney engineering is, not surprisingly, still at the stage of fairly crude demonstrations in culture and in animals [9,10].

There are three main approaches to tissue engineering. One approach places the greatest creative load on the engineers themselves, who create template structures by casting, electrospinning or 3D printing and then seed them with living cells [11,12]. So far, this has been most effective for simple structures. Another approach places the creative load on natural development and uses complex tissue structures from a deceased organ donor: a donated tissue or organ is cleared of cells by a method that preserves the extracellular matrix, and fresh cells (for example patient-derived stem cells) are placed in that ‘ghost’ matrix and so adhere to its complex anatomy. This method has seen clinical use for structures of moderate complexity such as trachea [13]. The third approach capitalizes on the self-organizing ability of many cell types, and the most realistic kidneys engineered to date have been made by bringing kidney-forming stem cells together and using drugs to suppress anoikis (a type of elective cell death) the cells organize themselves into a mini-kidney in much the same way that they would organize themselves during fetal life [14,15].

All of these methods have been designed to produce tissues that are as anatomically and physiologically normal as possible, although the first approach could in principle be used to make deliberately abnormal tissues. This goal that may be much more reasonable than it first sounds.

## The case for extending tissue engineering beyond the normal

Normal tissues have their limitations. They are ideally suited to replace a damaged or worn-out part in an anatomically typical body, but some bodies are unusual. People born with congenital abnormalities may require anatomically or physiologically customized engineered tissues to lead normal lives; people with autoimmune diseases may require physiological functions such as insulin production to be placed in safe cell types rather than immune-targeted cells; people with advanced artificial limbs or sense organs may require specialized interface tissues between natural nervous system and machine; people dependent, even temporarily, on *ex-corporo* life-support machines may benefit greatly from physiologically active ‘tissues’ housed in those machines; and even for conventional tissue engineering, synthetic niches that are well adapted to culture conditions may be very useful in growing stem cells and differentiating them towards a desired fate.

None of these custom ‘tissues’ are natural outcomes of our evolved developmental mechanisms, so their production requires deliberate interference with cells, their environment or both. For relatively simple examples that require custom anatomy but in which cell physiology is normal, the techniques of casting, spinning or printing matrix supports in the shape of the required anatomy may be enough. For structures beyond the limits of direct fabrication or beyond the limits of cells to navigate and colonize fabricated structures, and where cell physiology itself needs to be unusual, a directly synthetic biological approach will be needed.

## Self-organizing synthetic ‘tissues’: a feasible goal?

**Figure 1:**
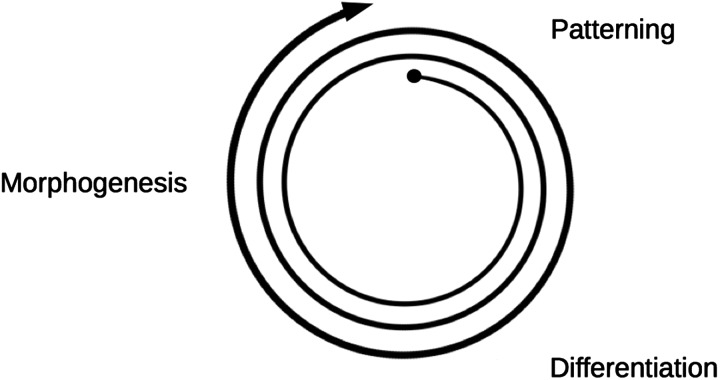
The embryological cycle Patterning directs differentiation which results in morphogenesis and the cycle repeats to add finer details as the embryo grows.

Normal embryonic development consists, in the main, of three processes that operate cyclically (Figure 1). One is patterning, which can create differences between cells that were identical, either *de novo*, as in the pattern of feathers on chick skin [16] or as a finer elaboration of an existing coarse pattern, as in the segmentation of the fruit fly [17]. A common result of patterning is cell differentiation, a stable change in gene expression according to the patterning signals. Some changes in gene expression drive the third process, morphogenesis, the creation of anatomical form. Morphogenesis, which for our purposes can be taken to include growth, creates differently shaped and sometimes larger fields of cells and, by folding of sheets and by cell migration, can bring distant cells into proximity. These processes can trigger new patterning events that in turn drive new differentiation and yet more morphogenesis. Thus, the three processes run in a loop, with finer and finer body details being added on each pass. Typically, the first pass through the loop generates very coarse body features (such as division into head and trunk), later iterations generate finer patterns (such as division of the trunk into segments, as demonstrated in our own bodies by the series of vertebrae), whereas later iterations still generate finer details such as the pattern of hairs on skin.

One approach to making truly synthetic tissues is to engineer artificial mechanisms for these processes; patterning, controlled gene expression and morphogenesis. The rest of this article will be devoted to reviewing early progress in this field. It will be seen that only the first, crude steps have yet been taken in this direction but that the results appear to be promising.

## Synthetic biological patterning mechanisms

**Figure 2:**
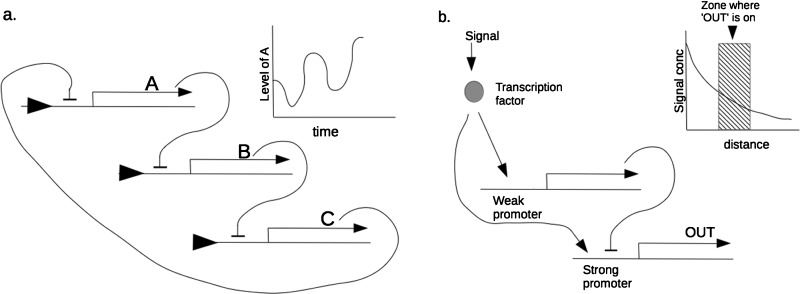
Synthetic biological modules for patterning in bacteria (**a**) Depicts the repressilator of Elowitz and Leibler [19], 2000 that generates oscillations, effectively a pattern in time; (**b**) depicts a module that interprets a gradient of signal concentration to generate a central ‘stripe’ only in zones of moderate signal concentration.

The first synthetic biological patterning systems built were oscillators that generated patterns in time rather than in space. This may seem to be unrelated to the spatial problems in tissues but, as it is possible to transform patterns in time into patterns in space and vice versa, they should be included. Indeed, even natural embryogenesis includes clock and wavefront models that combine a clock with a wave that sweeps along a tissue and leaves a ‘mark’ where the wave is whenever the clock reaches a particular phase. An example, albeit one with many refinements over the simple system just described, is vertebrate somitogenesis [18]. Conversely, a wave travelling along a tissue patterned with a spatial repeat such as stripes could, by triggering expression of a molecule only when the wave is crossing one colour; generate a temporal pattern from the spatial one. The first synthetic biological oscillator was the bacterial ‘repressilator’ [19], a cyclic sequence of three genes, each of which encoded a protein that would suppress transcription of the gene ahead of it in the cycle (Figure 2a). Subsequent refinements so synthetic oscillators have added synchronization between cells [4] or between populations of cells [20].

Some spatial patterning systems have also been built. The first ones were not capable of generating patterns *de novo*, but could at least elaborate a simple external cue into a more complex pattern. One example [21] interpreted a simple spatial gradient of signal concentration to create a stripe in the region of moderate concentration. It coupled detection of the signal to the activation of two genes: one, activated by an efficient promoter, drove transcription of the final output reporter whereas the other, activated by a relatively inefficient promoter, drove a repressor final output transcription (Figure 2b). In regions of very low concentration, there was too little signal to drive the transcription of the output gene. In regions of moderate concentration, there was enough to drive the efficient promoter of the output gene. In regions of very high concentration, even the inefficient promoter of the repressor gene was active, and transcription of the output gene was repressed. The gene was therefore only ‘on’ in the zone of moderate signal concentration. A similar network has been engineered in mammalian cells [22]. Again, refinements to this system have been made, and other patterning systems that take their cues from the edge of bacterial cultures, instead of manually-applied gradients, have been produced [23].

**Figure 3:**
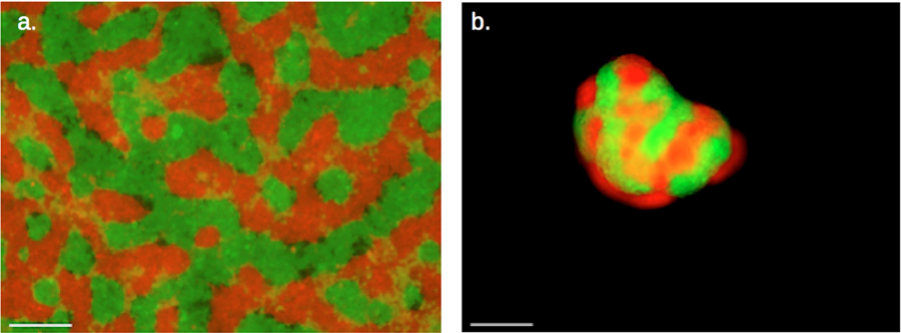
*De novo* pattern formation by cadherin-driven phase separation This is seen in (**a**) 2D and (**b**) 3D. Reproduced from [33]: Cachat, E., Liu, W., Martin, K.C., Yuan, X., Yin, H., Hohenstein, P. and Davies J.A. (2016) 2- and 3-dimensional synthetic large-scale *de novo* patterning by mammalian cells through phase separation. Sci. Rep., doi:10.1038/srep20664.

Patterning fields of cells *de novo*, with no pre-existing cues, is more of a challenge. Attempts are underway to produce a synthetic version of patterning by reaction–diffusion mechanisms thought to operate in real embryos [24,25] but, at the time of writing, no working system seems to have published although some promising tools already exist [26]. Other efforts to generate *de novo* patterning used orthogonal control of cell motility to establish stripes on 2D lawns [27]. Others have created synthetic circuits that could be used to generate lateral-inhibition patterns in mammalian cells, with components of the Notch–Delta signalling pathway [28,29]. We have taken a different approach to building a *de novo* patterning system that uses adhesion-driven phase separation which is not, as far as we know, widely used by embryos. The system operates by cells expressing one of two types of calcium-dependent adhesion molecules, E-cadherin or P-cadherin. Cells carrying these proteins behave as if E-cadherin binding to E-cadherin reduces free energy (adheres) somewhat more than P-cadherin to P-cadherin, but both reduce free energy much more than E-cadherin to P-cadherin contacts [30]. It has been known for many years that mixtures of low numbers of cells carrying these cadherins sort into homogeneous groups to maximize homotypic contacts and minimize energetically unfavourable heterotypic ones [31]. This complete separation depends on the system not becoming trapped in a local energy minimum. Our computer modelling suggested that large numbers of cells would become trapped in a local minimum, forming stripes or spots (depending on cell ratios) instead of separating completely. Constructing the system in a human cell line has confirmed this behaviour, in both 2D and 3D culture systems (Figures 3a and 3b). The next challenge for this system will be to refine it and add a second-pass, elaboration stage to make a more detailed pattern.

## Synthetic biological morphogenetic mechanisms

The ‘developmental cycle’ (Figure 1) leads from patterning through changes in gene expression to morphogenesis. An analysis of morphogenetic processes, in an early speculative paper about prospects for synthetic morphogenesis [32], suggested that most examples of mammalian morphogenesis use combinations of approximately ten basic morphogenetic events. These are cell proliferation, cell death, cell fusion, cell adhesion, cell de-adhesion, cell migration, epithelial-to-mesenchyme transition, mesenchyme-to-epithelial transition, epithelial folding and lumen formation. In principle, being able to invoke these events through changes in gene expression following patterning would allow the construction of an artificial ‘developmental cycle’.

Fortunately, a range of past studies had identified certain genes (some of them from non-embryological sources, such as viruses), the activation of which can drive a specific one of the ten basic morphogenetic events. This observation has allowed us to produce a set of modules for ‘synthetic morphology’, that allow control of proliferation, elective cell death, cell fusion, cell adhesion and locomotion [5]. These have been published separately from work on patterning but, in principle, morphogenetic modules can be placed downstream of patterning. A simple proof-of-principle example has been made, in which patterning by phase separation is followed by triggering of a cell death module in one colour of cells to transform the pattern of colours into a net-like arrangement of cells and spaces.

## The next steps

Clearly, the demonstrations described above, although they do show the feasibility of synthetic biological systems for patterning and morphogenesis, are a long way from being useful synthetic tissues. More patterning systems are clearly needed, and they need to be enriched with responsive systems so that patterning can be guided with respect to outside cues. The morphogenetic systems are currently limited to simple 2D demonstrations; additional modules to allow cells to make multilayered assemblies are urgently needed, as are modules that encourage interaction of boundary layers with specific normal body cells. In addition, modules that encourage interaction with machinery, for *ex-corporo* applications, would be useful especially as *ex-corporo* applications are likely to be the first to be translated to actual use as the safety implications are much less troubling than they would be for *in vivo* use.

Classical tissue engineering will continue as synthetic biology develops towards being useful to it and most of the current priorities for tissue engineering, which all centre on normal tissues, may probably achieved without a synthetic input. Synthetic biological approaches may, however, make the task easier. One of the most promising techniques for classical tissue engineering is bioprinting, in which living cells are laid down already embedded in their 3D matrix [34]. Bioprinting has, for example been used to produce 3D alginate-based matrices containing living human induced pluripotential stem (iPS)-derived hepatocyte-like cells or iPS cells that were differentiated into hepatocyte-like cells *in situ* [35]. Many printing processes, however, place serious stresses on cells due, depending on the technique (e.g. inkjet, extrusion, laser-assisted [34], to heat, shear stress, impact and vibration). The need to control these variables is a serious limit on the printing technologies that can be used, forcing sacrifice of the conditions optimal for printing for the sake of keeping stresses low. In general, methods have to be optimized for each application and, even then, viabilities can be in the region of 60% for differentiated cells [35] to 90% for embryonic stem (ES) cells [36]. One possible use for synthetic biology would be to confer on cells an inducible state that makes them more tolerant of printing, for example by altering the cytoskeleton to make it more flexible (red blood cells, for example have cytoskeletons that make then very tolerant of shear) and expressing antioxidants and heat-shock proteins. Even if this cannot be done, having the cells report their levels of stress in way that can be detected by the printer (e.g. fluorescence) might allow the use of closed-loop control in which the printing machine will operate in a gentler mode when this becomes necessary. A second application might be to confer on cells superior binding to conveniently-printed materials such as alginate, because the currently used technique of supplementing these artificial materials with natural animal matrix components such as collagen can complicate the printing process [34].

The other major method of tissue engineering eschews bioprinting and instead uses cells' own ability to organize themselves into organoids [37–39]. As has already been noted, the structures formed tend to be limited to those that resemble existing tissues, the construction involving changes of gene expression in sequences similar to those seen in normal development in a process that has been termed *genetically encoded self-assembly* [40]. A hybrid approach would be to add synthetic biological features to the existing genetic programme so that self-assembly is altered. This might be achieved by altering cell motility or adhesion [5]. More spectacularly and flexibly, it has proved possible to modify cell surfaces so that they carry DNA strands, and then to use substrates printed with complementary strands so that each cell type adheres precisely where it is required [41]. In principle, it may be possible to alter preferences of cell–cell adhesion in this way, even without a matrix (the DNA on some cell surfaces being complementary to that on other cells). By methods such as this, different cells could be organized a specific way before they begin their own ‘evolved’ self-organizing programmes, providing a more predictable location of key features.

If development of synthetic biological tools for patterning and morphogenesis continues and expands then, by the time tissue engineering is ready to meet the challenges of producing non-natural tissues, the required technologies may be in place. It must be noted, though, that synthetic biologists entering the field from the prokaryotic world might find mammalian systems frustrating. To begin with, they are slow, generation times being of the order of a day rather than tens of minutes. Introduced genes are also subject to epigenetic effects, particularly gene silencing through chromatin modification. Finally, the natural cell–cell interactions that are so critical to normal development and to organoid formation will still be up-and-running, unless the synthetic systems actively block them. Given our still-imperfect knowledge of development, these signals and the cells’ responses to them, become difficult to predict when cell relationships have been altered by tissue engineering. Mammalian synthetic biology remains a challenge–hopefully, a challenge that will prove very attractive to new bioengineers keen to prove themselves.

## Abbreviations

ES cell: embryonic stem cell
iPS cell: induced pluripotential stem cell
